# Genome-wide association study (GWAS) with high-throughput SNP chip DNA markers identified novel genetic factors for mesocotyl elongation and seedling emergence in rice (*Oryza sativa* L.) using multiple GAPIT models

**DOI:** 10.3389/fgene.2023.1282620

**Published:** 2023-11-20

**Authors:** Nkulu Rolly Kabange, Simon Alibu, Youngho Kwon, So-Myeong Lee, Ki-Won Oh, Jong-Hee Lee

**Affiliations:** ^1^ Department of Southern Area Crop Science, National Institute of Crop Science, RDA, Miryang, Republic of Korea; ^2^ National Crops Resources Research Institute (NaCRRI), National Agricultural Research Organisation (NARO), Entebbe, Uganda

**Keywords:** mesocotyl, emergence, GWAS, GAPIT, SNP chip DNA markers

## Abstract

This study employed a joint strategy high-density SNP Chip DNA markers and multiple Genome Association and Prediction Integrated Tool (GAPIT) models [(Bayesian-information and Linkage-disequilibrium Iteratively Nested Keyway (BLINK), Fixed and random model Circulating Probability Uniform (FarmCPU), General Linear Model (GLM), and Settlement of Mixed Linear Model (MLM) Under Progressively Exclusive Relationship (SUPER)], to investigate novel genetic factors controlling mesocotyl elongation and seedling emergence for direct-seeded rice. Genotype data (230,526 SNP Chip DNA makers) of 117 doubled haploid lines (derived from a cross between 93–11 (*Oryza sativa* L. ssp. *indica*) and Milyang352 (*O. sativa* L. ssp. *japonica*) were used to perform a Genome-Wide Association Study (GWAS). Results revealed the association between five (5) topmost significant SNP markers, of which number two [AX-155741269, Chr2: 15422406 bp, and AX-155200917, Chr7: 23814085 bp, explaining 37.5% and 13.8% of the phenotypic variance explained (PVE)] are linked to the mesocotyl elongation loci, while three (AX-282097034 and AX-283652873, Chr9: 9882817 bp and 1023383 bp, PVE 64.5%, and 20.2%, respectively, and AX-154356231, Chr1: 17413989 bp, PVE 21.1%) are tightly linked to the loci controlling seedling emergence. The *qMEL2-1* and *qSEM9-1* are identified as major QTLs explaining 37.5% and 64.5% of the PVE for mesocotyl elongation and seedling emergence, respectively. The AX-282097034 (Chr9: 9882817 bp) was co-detected by four GAPIT models (BLINK, FarmCPU, SUPER, and GLM), while AX-155741269 was co-detected by BLINK and SUPER. Furthermore, a high estimated heritability (Mesocotyl elongation: h^2^ = 0.955; seedling emergence: h^2^ = 0.863; shoot length: h^2^ = 0.707) was observed. Genes harbored by *qMEL2-1* and *qSEM9-1* have interesting annotated molecular functions that could be investigated through functional studies to uncover their roles during mesocotyl elongation and seedling emergence events in rice. Furthermore, the presence of genes encoding transcription factors, growth- and stress response, or signaling-related genes would suggest that mesocotyl elongation and seedling emergence from deep direct-seeded rice might involve an active signaling cascade and transport of molecules, which could be elucidated through functional analysis. Likewise, genomic selection analysis suggested markers useful for downstream marker-assisted selection (MAS).

## Introduction

The successful establishment of plants in the field largely depends on the ability of dry seeds to germinate, emerge, and fully differentiate. Failure in the emergence of seedlings may lead to crop failure and yield loss. Direct seeding is a common agricultural practice in many upland and lowland rice cultivation systems and helps save water and reduces labor costs (Kumar and Ladha, 2011; Jang et al., 2021). This agricultural practice is categorized as wet and dry direct seeding. Wet direct seeding refers to pre-germinated seeds being sown on wet or puddled soil. In contrast, dry direct seeding denotes the condition where seeds are drilled on unpuddled soil after dry tillage (Kumar and Ladha, 2011). In general, wet direct seeding is practiced in lowland rice cultivation environments, while dry direct seeding is commonly suitable for upland rice cultivation systems. Nevertheless, due to the variable seeding depths, and in some cases sub-optional seedbed conditions, direct seeding is often prone to poor seedling emergence and establishment. Consequently, a reduction in the number of individuals in the field is observed, leading to reduced yield and production (Alibu et al., 2012; Romaneckas et al., 2022). Seeds are sown at 5–10 cm depth, and their emergence after germination could be affected by factors such as soil texture and moisture, seed vigor or genetic makeup, and physiological and biochemical properties of seeds. Advanced statistical tools applied in plant breeding and genome sequencing offer new possibilities to gain new insights into the genetic control of agronomically important traits at the whole genome level.

In addition, poor leveling and uneven distribution of water in paddy fields lead to deep spots that affect the ability of wet-seeded rice to germinate and elongate due to hypoxia; therefore resulting in poor emergence and establishment (Hossain & Teixeira da Silva, 2013). Ideally, wet-seed lowland rice should be sown at a seeding depth of approximately 2.5 cm under wet soil. In upland rice, however, 2‒4 cm is recommended. The germination and emergence of upland rice sown at 2‒4 cm depth is generally affected by short dry spells that occur shortly after sowing and dry out the topsoil on which the seed is placed. Therefore, deep seeding may be necessary for direct dry-seeded upland as it can benefit from residual soil moisture in the deeper layers of soil, improving germination and emergence (Romaneckas et al., 2022). For that reason, rice accessions recommended for dry-direct seeding are required to have the ability to withstand deep sowing or tolerate variable seeding depths associated with machine sowing (Zhao et al., 2018).

The mesocotyl is a crucial agronomic trait for direct seeding because it can enhance seedling emergence and establishment from deep sowing (Lu et al., 2007; Zhan et al., 2020; Menard et al., 2021), and rice genotypes that rapidly elongate their mesocotyls are desirable for dry direct seeding (Alibu et al., 2012). The mesocotyl is a tubular, white stem-like tissue connecting the seed and the base of the coleoptile. Technically, the mesocotyl is the first internode of the stem. After germination, elongation of the mesocotyl elevates the coleoptile and its enclosed inner leaves towards the soil surface, thereby enhancing seedling emergence from deep sowing (Zhan et al., 2020; Menard et al., 2021). As the coleoptile nears the soil surface, exposure to light triggers dynamic endogenous changes in the phytohormone levels, which immediately halts mesocotyl elongation (Feng et al., 2017). Thus, rice cultivars that rapidly elongate their mesocotyls are desirable for direct seeding.

Mesocotyl elongation is a genetically determined trait that is measurable and controlled by the cumulative effect of many genes, influenced by environmental factors such as light, temperature, and water, and is responsive to sowing depth, soil water content, and salinity. Furthermore, plant hormones such as abscisic acid (ABA), Brassinosteroids (BR), strigolactone (SLs), cytokinin (CK), ethylene (ETH), jasmonic acid (JA), gibberellin (GA), and indole-3-acetic acid (IAA) play important roles in regulating mesocotyl elongation (Zhan et al., 2020). Mesocotyl elongation varies among cultivars of different rice groups. For example, *indica* rice accessions have been shown to exhibit longer mesocotyls when compared to the Japonicas (Uzair et al., 2022). Between *indica* and *japonica* rice, *indica* exhibited the longest mesocotyls with the highest length variation (Menard et al., 2021). In general, *indica* rice varieties exhibit longer mesocotyls with a larger variation than *japonica* rice.

In a study conducted by Zhao et al. (2018), 46.7% of *japonica* accessions showed shorter mesocotyls (0–1 cm) than their *indica* counterparts (30.2 %). However, more *indica* accessions had long mesocotyls of 4 cm or longer than in *japonica*. Likewise, Ming-guo et al. (2005) observed that an upland rice variety showed longer mesocotyls than lowland rice. Similarly, rice accessions originating from South and Southwest Asia assessed for their mesocotyl elongation patterns showed a larger variation than those originating from East Asia (Takahashi, 1978). This large natural genetic variation in mesocotyl elongation provides a basis for developing long mesocotyl cultivars (Zhan et al., 2020) and identifying genes by linkage mapping and/or Genome-Wide Association Study (GWAS) (Zhan et al., 2020). A large number of QTLs controlling rice mesocotyl elongation have been identified using different genetic populations, including recombinant inbred lines (RILs), chromosome segment substitution lines (CSSLs), backcross recombinant inbred lines (BILs) and doubled haploid (DH) populations (Zhan et al., 2020).

The advent of genome sequencing and advances in molecular breeding techniques, coupled with the emergence and application of bioinformatics (for gene discovery, GWAS, or genomic selection (GS) among others) have boosted the efficiency of plant breeding and variety development in a relatively short period. The Genome Association and Prediction Integrated Tool (GAPIT) package in R software is widely used in several genome-wide association studies and has successfully generated an abundance of data useful in plant breeding. Since it first launch of the first version in 2012, two more versions of the GAPIT package have been released, and the latest version 3 with multiple statistical models boosted the power and accuracy of GS and prediction (Wang et al., 2021). At the same time, these technologies and tools have offered tremendous opportunities for scientists to uncover novel genetic factors associated with desired and important traits in plant crops. The Single Nucleotide Polymorphism (SNP) marker system gained momentum in plant bioscience (Mammadov et al., 2012). Likewise, high-throughput SNP genotyping and DNA chip technology have emerged as powerful genomic tools in plant bioscience-related research to accelerate crops improvement (Thomson and Biotechnology, 2014; You et al., 2018; Yu et al., 2021; Carrier et al., 2022). Recently, with the rapid development of molecular marker technology, several quantitative trait loci (QTLs) for mesocotyl elongation were identified using bi-parental linkage mapping in rice (Lu et al., 2007). More than 40 mesocotyl elongation QTLs across almost all 12 chromosomes have been reported in rice using various segregating lines. Identification of QTLs associated with mesocotyl length could accelerate the genetic improvement of rice for wet and dry direct seeding.

This study aims to investigate novel QTLs controlling mesocotyl elongation and seedling emergence of direct-seeded rice through a GWAS with high-throughput SNP Chip DNA markers, using multiple GAPIT models with enhanced statistical power. The study also, capitalized on the multiple GAPIT to conduct a GS for the traits of interest.

## Materials and methods

### Plant materials and growth conditions

A hundred seventeen (117) Doubled haploid (DH) lines developed through anther culture of the cross between 93–11 (*Oryza sativa* L. ssp. *indica*) and Milyang352 [*O. sativa* L. ssp. *japonica* (Lee et al., 2020)]. Milyang352 is a Korean cultivar earlier developed from a cross between a Chinese rice cultivar C18 and Ungwang (Korean cultivar). Initially, seeds of the parents and DH lines were soaked for 3 days to induce germination. Thereafter, 20 seeds per line were sown on about a 2-cm soil bed and covered with a 6-cm soil layer (depth) in a seed box with compartments. The phenotype parameters {mesocotyl length, seedling emergence percentage [(number of emerged seedlings/total number of seeds sown) ×100], and shoot length} were recorded 14 days after sowing.

### Frequency distribution, quantile‒quantile plots, kinship matrix, and correlation analysis

The frequency distribution and the correlation between traits were assessed using GraphPad Prism 7.0. The heat map was generated after installing [install.packages (“*package name*”)] and executing the library [library (“*package name*”)] of *R* packages *ggplot2*, *tidyverse*, *cluster*, *factoextra*, and executing the following *R* scripts:

[Script 1]: gr <- colorRampPalette [c (“green,” “red”)]
[Script 2]: heatmap.2 [y,col = gr, main = “Input main title here,” trace = “none,” margins = c (10,12), cexRow = 0.5, cexCol = 0.9, scale = “column,” srtCol = 0, adjCol = c (1,1), tracecol = NA, reorderfun = function (d, w) reorder (d, w, agglo. FUN = mean)].

The Quantile‒Quantile (Q‒Q) plots and the pairwise kinship matrix were generated after installing and executing the GAPIT function for GWAS. Meanwhile, the principal component analysis was done using the *R* package *ggplot2* [*install.packages* (*“ggplot2″*)] as follows:

install.packages (“ggplot2”)
library (ggplot2)
Dataset <- read. csv (“C://data file location pathway. csv,” sep = “,”)
data <- data. matrix (Dataset)
head (data)
myPr <-prcomp [data (,−1), scale = TRUE]
myPr
summary (myPr)
plot (myPr, type = “l”) #Generate a scree plot
biplot (myPr, scale = 0) #Generate a PCA plot

### Genomic selection or prediction analysis

To assess the genetic merit of the DH lines for target traits, we conducted a genomic prediction analysis as described earlier (Zhang et al., 2007). For GS analysis, we used the mixed linear model (MLM) (Yu et al., 2006), also known as genomic best linear unbiased prediction (gBLUP), which has a higher prediction accuracy for traits controlled by a large number of genes.

The genotype data was converted from the Haplotype Map (HapMap) format to numerical (see *R* script below) prior to performing the analysis. Together with the phenotype data, genotyping data were used to conduct a GP in order to assess the genomic estimated breeding value (GEBV) of the DH population. The GS analysis was done using the gBLUP method to estimate the genomic breeding values of the mapping population.

*To convert HapMap to numerical format*:

myG <-fread (“file:///D:genotype data location. txt,” head = FALSE)
myGAPIT <- GAPIT (G = myG, output. numerical = TRUE)
myGD = myGAPIT$GD
myGM = myGAPIT$GM
To conduct a genomic prediction:
myY < -read.csv (“phenotype file location pathway. csv,” sep = “,”)
myGD = read.csv (“numerical genotype file location pathway. csv,” sep = “,”)
myGM = read.csv (“markers file location pathway. csv,” sep = “,”)
set.seed (99,163)
GAPIT.Validation [
Y = myY (,1:2),
model = c (“gBLUP”),
GD = myGD, GM = myGM,
PCA.total = 3,
file.output = T,
nfold = 5]

The genomic prediction of the inference groups, which is based on the ties with corresponding groups in the reference panel, is derived from Henderson’s formula as follows:

*u*_I_ = K_IR_K_RR_^–1^ *u*_R_, where K_RR_ is the variance-covariance matrix for all groups in the reference panel, K_RI_ is the covariance matrix between the groups in the reference and inference panels, K_IR_ is the covariance matrix between the groups inference and reference panels, *u*_R_ is the predicted genomic values of the individuals in the inference group. To assess the reliability of the genomic prediction, the following formula is used:

*Reliability= 1–PEV/*σ^*2*^_*a*_, where PEV is the prediction error variance, representing the diagonal element in the inverse left-hand side of the mixed model equation, and σ^2^_a_ is the genetic variance.

### Genome-wide association study

To investigate genetic factors associated with the control of mesocotyl elongation and seedling emergence in rice, 230,526 markers were used. A GWAS was run by GAPIT (Genome Association and Prediction Integrated Tool) version 3 (Wang et al., 2021) with multiple models. The GAPIT models included the Bayesian-information and Linkage-disequilibrium Iteratively Nested Keyway (BLINK) (Zhang et al., 2018), Fixed and random model Circulating Probability Uniform (FarmCPU) (Liu et al., 2016), General Linear Model (GLM) (Price et al., 2006), Mixed Linear Model (MLM), and Settlement of MLM Under Progressively Exclusive Relationship (SUPER) (Wang et al., 2014), were employed to perform the genomic association analysis in R programming software. *R* scripts proposed by:

To perform a GWAS analysis:

my_GAPIT <- GAPIT [Y = myY, G = myG, model = c (“SUPER,” “FarmCPU,” “BLINK”), PCA. total = 3, SNP. MAF = 0.05, Multiple_analysis = TRUE].

### In silico analysis and gene ontology search

GWAS analysis provided useful information on major QTLs for mesocotyl elongation and seedling emergence in rice. Based on physical positions of associated significant SNP Chip markers, we unraveled the identity of genes found within the target genetic loci to gain more insights. To achieve that, we conducted a search using the browser of the Rice Genome Annotation Project database (http://rice.uga.edu/cgi-bin/gbrowse/rice/#search, accessed on 27 July 2023) and PlantPAN 3.0 (http://plantpan.itps.ncku.edu.tw/plantpan3/search.php?#results, accessed on 27 July 2023) for each specific gene locus ID. Genes encoding similar domain-containing proteins were searched in the literature (https://funricegenes.github.io/geneKeyword.table.txt, accessed on 27 July 2023).

To assess the similarity of target genes in *qSEM9* and *qMEL2* between *japonica* and *indica* groups, the coding sequences (CDS) of putative candidate genes, obtained from the Nipponbare rice database (http://rice.uga.edu/#search, accessed on 13 October 2023) and *indica* rice database (https://plants.ensembl.org/Oryza_indica/Info/Index, accessed on 13 October 2023), were aligned using the ClustalW algorithm in BioEdit software (BioEdit Sequence Alignment Editor, Copyright ^©^1997–2013 Tom Hall) (Hall, 1999).

### Total RNA extraction, cDNA synthesis, and qPCR analysis

Total RNA was extracted from leaf, roots, and mesocotyl of two-week-old seedlings, following the manufacturer’s instructions, using the RNeasy Plant Mini Kit (Qiagen, Netherlands). In essence, approximately 50–100 mg of frozen samples in liquid nitrogen were ground to a fine powder in 2 mL Eppendorf tubes (e-tubes) using beads by vigorous vortex. Then 450 µL buffer RLT (lysis buffer containing β-mercaptoethanol) was added, and the tubes were vigorously vortexed. The lysate was transferred to a QIAshredder spin column (lilac) placed in a 2 mL collection tube, followed by centrifugation for 2 min at 12,000 rpm. The supernatant of the flow-through was transferred to a new microcentrifuge tube (1.5 mL). The clear lysate was mixed with 0.5 volume of ethanol by pipetting up and down for a few seconds, and about 650 µL of the sample mixture was transferred to an RNeasy Mini spin column (with a membrane) with a 2 mL collection tube. The tubes were centrifuged for 15 s at 10,000 rpm. The flow-through was discarded and 500 µL of Buffer RPE was added to the RNeasy spin column, followed by centrifugation at 10,000 rpm for 2 min. To elute the RNA, 50 µL RNase-free water was added to the membrane of the RNeasy spin column, and the tubes were centrifuged for 1 min at 10,000 rpm. The concentration RNA (ng µL^–1^) was measured using a NanoDrop2000 Spectrophotometer (Thermo Fisher Scientific, USA), and samples were kept in −20°C fridge for downstream analysis.

For complementary DNA (cDNA) synthesis, the ProtoScript II First cDNA Synthesis Kit (New England BioLabs Inc., USA) was employed. Briefly, 1 µg RNA samples was preheated with 1 µL Oligo dT at 65°C for 5 min. Thereafter, 2 µL ProtoScript II Enzyme Mix (10X) was added, and the reaction mixture was adjusted to 20 µL reaction volume with RNase-free water. The cDNA synthesis reaction was incubated at 42°C for 1 h in a Reverse transcriptase PCR using a Thermocycler.

To validate the expression of genes found within *qSEM9* and *qMEL2*, qPCR analysis was performed using the amfiSure qGreen Q-PCR Kit (GenDEPOT Corporation ^©^2017, USA) in a (QuantStudioTM real-time PCR systems, Thermo Fisher Scientific, USA). qPCR machine. In essence, a three-step reaction consisting of an initial enzyme activation at 95°C for 3 min, followed by 40 cycles of denaturation at 95°C for 10 s, annealing 60°C for 20 s, and extension at 72°C for 30 s.

## Results

### Differential mesocotyl length and seedling emergence between parental lines

**FIGURE 1:**
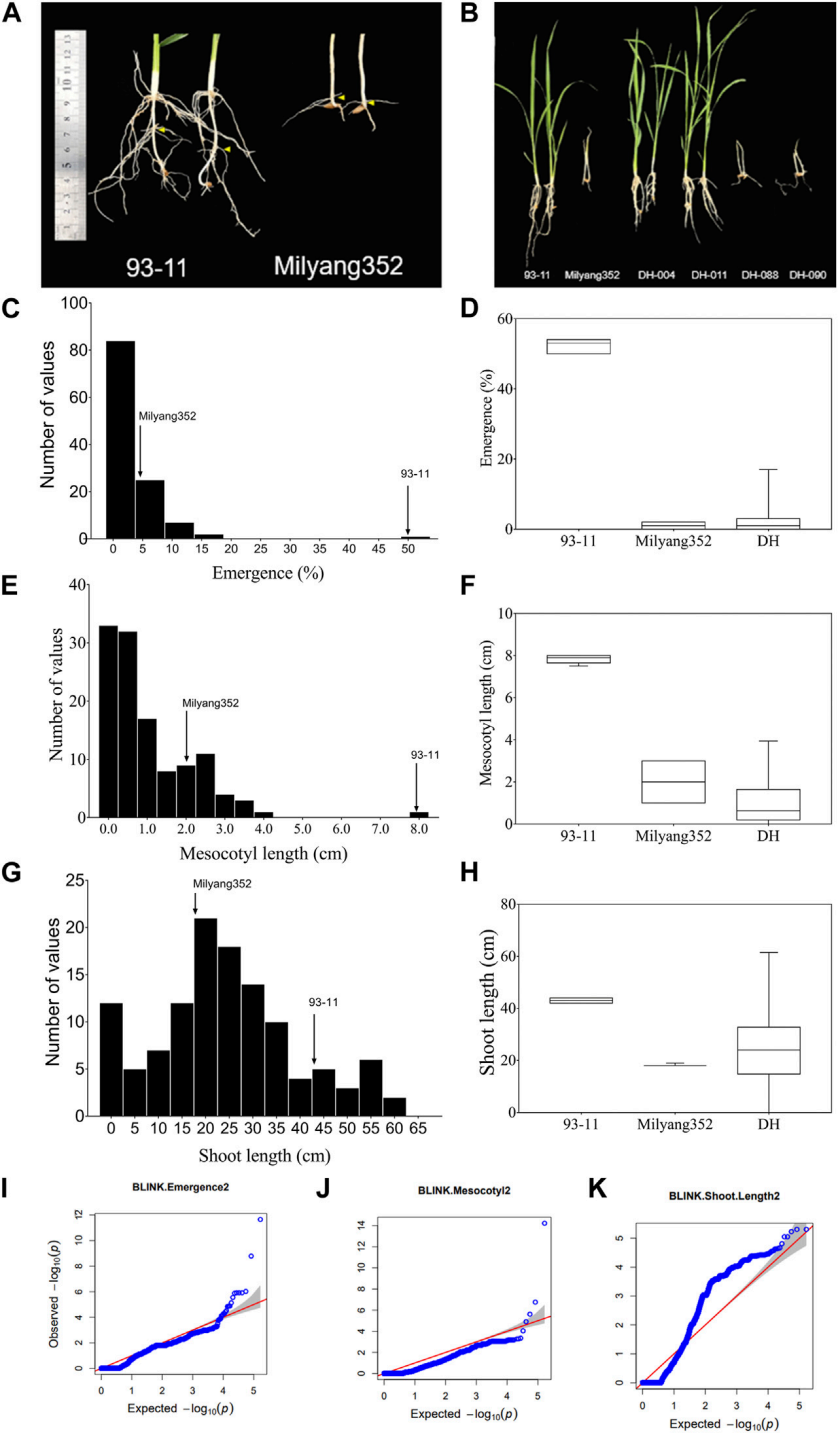
Frequency distribution of traits and quantile–quantile plots. **(A,B)** Mesocotyl length and shoot emergence pattern of parental lines (93–11 and Milyang352) and a few DH lines, **(C–H)** Frequency distribution and box plots for seedling emergence, mesocotyl length, and shoot length of 117 rice doubled haploid lines. **(I–K)** Quantile–Quantile (Q–Q) plots for seedling emergence, mesocotyl length, and shoot length. –log10(*p*) is the negative logarithm base 10 quantile–quantile (Q–Q) of the *p*-values (expected and observed) for traits.

We observed differential mesocotyl elongation and shoot emergence patterns between parental lines 14 days after sowing. Results indicate that the *indica* parent (P1, 93–11) developed longer mesocotyl (7.9 cm) and taller seedlings (43.4 cm) (compared to its *japonica* counterpart (P2, Milyang352; mesocotyl length: 2 cm, shoot length: 18 cm) (Figures 1A, F). Likewise, the emergence percentage (here expressing the number of seedlings that emerged at the last count) was much higher in 93–11 (on average 50%) than that recorded in Milyang352 (on average 5%) (Figures 1A, B, D). The poor emergence of Milyang352 resulted in poor growth and shoot elongation (Figures 1A, B, H). Under the same conditions, about 74.4% of the mapping population (117 doubled haploid lines) showed a mesocotyl elongation pattern similar to that of Milyang352, while 25.6% resembled the mesocotyl elongation of 93–11-like. Likewise, nearly 84.6% of the DH lines had a Milyang352-like emergence percentage against 15.4% with a 93–11-like emergence pattern (Figures 1A, B, D, F, H). In addition, 67.5% of the DH lines recorded relatively taller seedlings, while 32.5% had shorter seedlings similar to 93–11 or Milyang352, respectively. Furthermore, panels Figures 1C, E indicate a positive (right) skewness distribution for seedling emergence and mesocotyl elongation, while Figure 1G shows that DH lines exhibited a normal distribution for shoot length. Furthermore, the quantile-quantile (Q–Q) plots show the distribution of data to the theoretical values for the traits of interest (Figures 1I–K).

### Cluster analysis, principal component analysis, and correlation between traits

**FIGURE 2:**
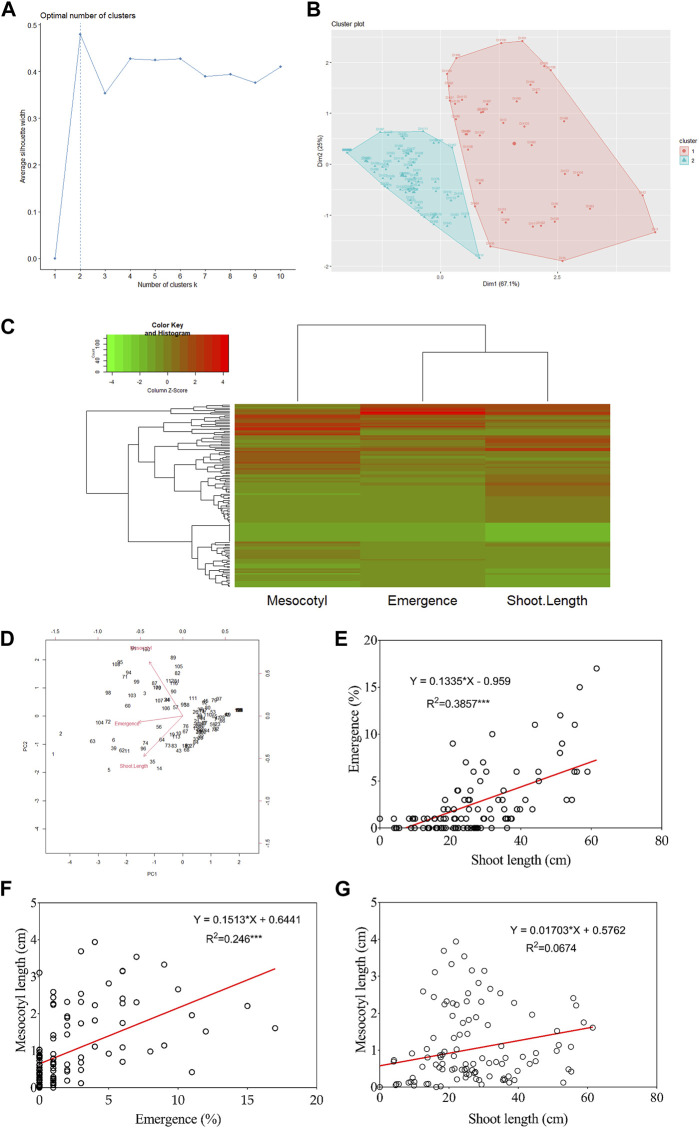
Cluster, principal component analysis, and correlation results. **(A)** Scree plot defining the number of clusters of the population, **(B)** Population clustered into two clusters, **(C)** Heat map with groups of DH lines based on phenotypes, **(D)** Principal Component Analysis results, and **(E–G)** Correlation analysis results between traits.

Figures 2A, B show that DH lines were grouped into two distinct clusters based on their mesocotyl elongation pattern (93–11 or Milyang352-type). From another perspective, the data in panel Figure 2C (heat map) groups seedling emergence and shoot length in the same cluster, while mesocotyl elongation is assigned to a different cluster (column dendrogram on top). Panel Figure 2C also allows visualizing the respective phenotypic pattern of all DH lines (lines with a high phenotypic values are shown by the red color map, while those with low phenotypic values are indicated in green). In addition, the principal component analysis (PCA) proposes that PC1, PC2, and PC3 explained 75.1% and 80% of the proportion of variance for seedling emergence and mesocotyl elongation, respectively (Figure 2D). We were also interested to see the relationship between traits; we assessed the correlation of all traits. Data in Figure 2E indicate an existing strong positive correlation between seedling emergence and shoot elongation (*R* ^2^ = 0.386***). Similarly, mesocotyl elongation and seedling emergence exhibited a positive relationship (*R* ^2^ = 0.246***) (Figure 2F). In contrast, no correlation was found between mesocotyl elongation and shoot length (*R* ^2^ = 0.067) (Figure 2G).

### Co-ancestry, marker density, heritability, and genome prediction

**FIGURE 3:**
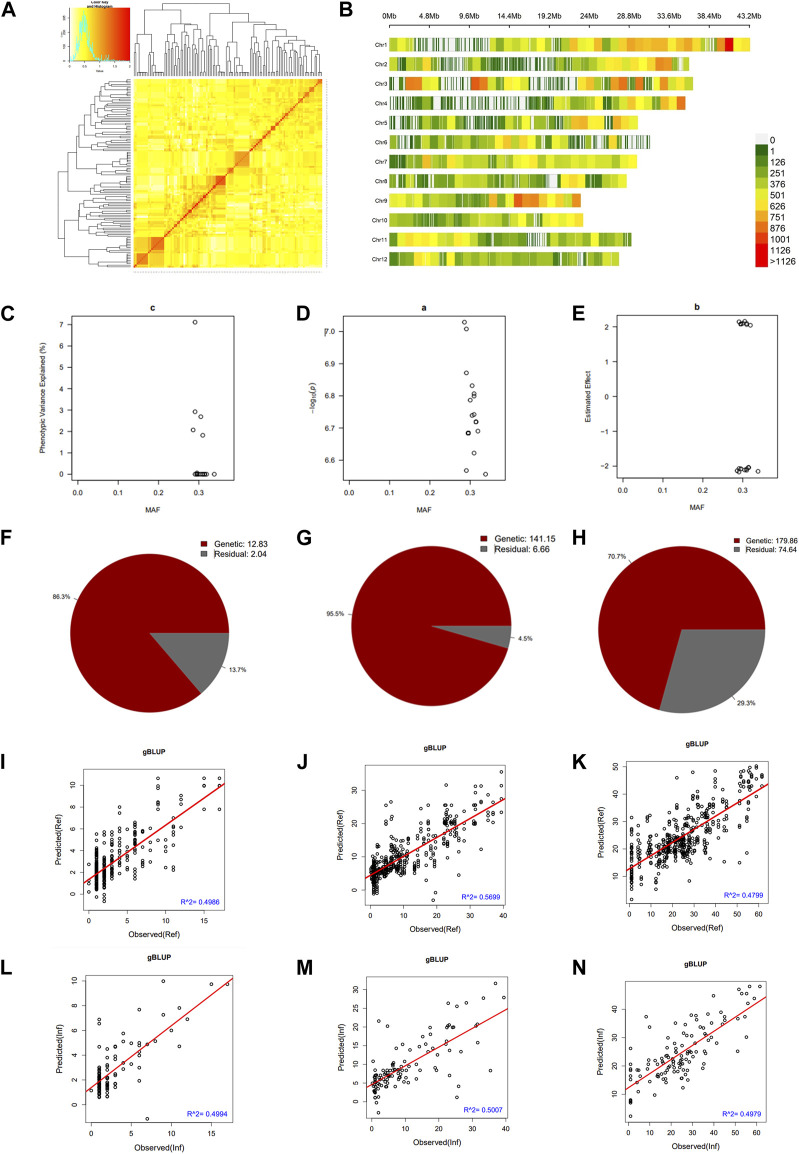
Pairwise kinship matrix, marker density map, genomic selection, and heritability results. **(A)** Pairwise kinship matrix showing the relatedness or co-ancestry level, **(B)** Density map of the markers per chromosome, **(C)** Phenotypic variance explained (PVE), **(D)** logarithm of the odds [LOD, –log10(*p*)] values, **(E)** estimated effect of the significant markers, **(F–H)** narrow sense heritability (h^2^) for mesocotyl elongation, seedling emergence, and seedling height, respectively, **(I–K)** predicted versus observed genomic estimated breeding value in the reference group and **(L–N)** in the inference group.

We constructed a kinship matrix to assess the population’s relatedness. Results in Figure 3A show the SNP Chip DNA marker-based kinship, also referred to as the co-ancestry or half relatedness. This data reveals the distribution of coefficients of co-ancestry of DH lines, confirming their genetic variability. In addition, Figure 3B displays the density map of used SNP Chip markers on all 12 chromosomes of rice. The phenotypic variance explained (PVE %) of significant SNP Chip, the logarithm of the odds (LOD) values, expressed as–log_10_(*p*), and the estimated effects are shown in panels Figures 3C–E.

We estimated the heritability of target traits to understand the proportion of variation explained by the individuals’ breeding values for each trait. Results in panels Figures 3F–H reveal that mesocotyl elongation had a heritability (h^2^) of 0.863, while seedling emergence and shoot length recorded an h^2^ of 0.955 and 0.707, respectively. To further gain insights, we used the GAPIT models to conduct a GS based on MLM (gBLUP), which is known to have a higher prediction accuracy to assess the genomic estimated breeding value (GEBV) of individuals for traits controlled by a large number of genes. Therefore, using this method, we investigated the genetic merit of DH lines for the target traits. Results in Figures 3I–N show the predicted and observed GEBV of individuals in the mapping population for mesocotyl elongation and seedling emergence, in the reference group (Ref) (Figures 3I–K) and the Inference group (Inf) (Figure 3L–N).

### Detected genetic loci controlling mesocotyl elongation and seedling emergence by GWAS

**FIGURE 4:**
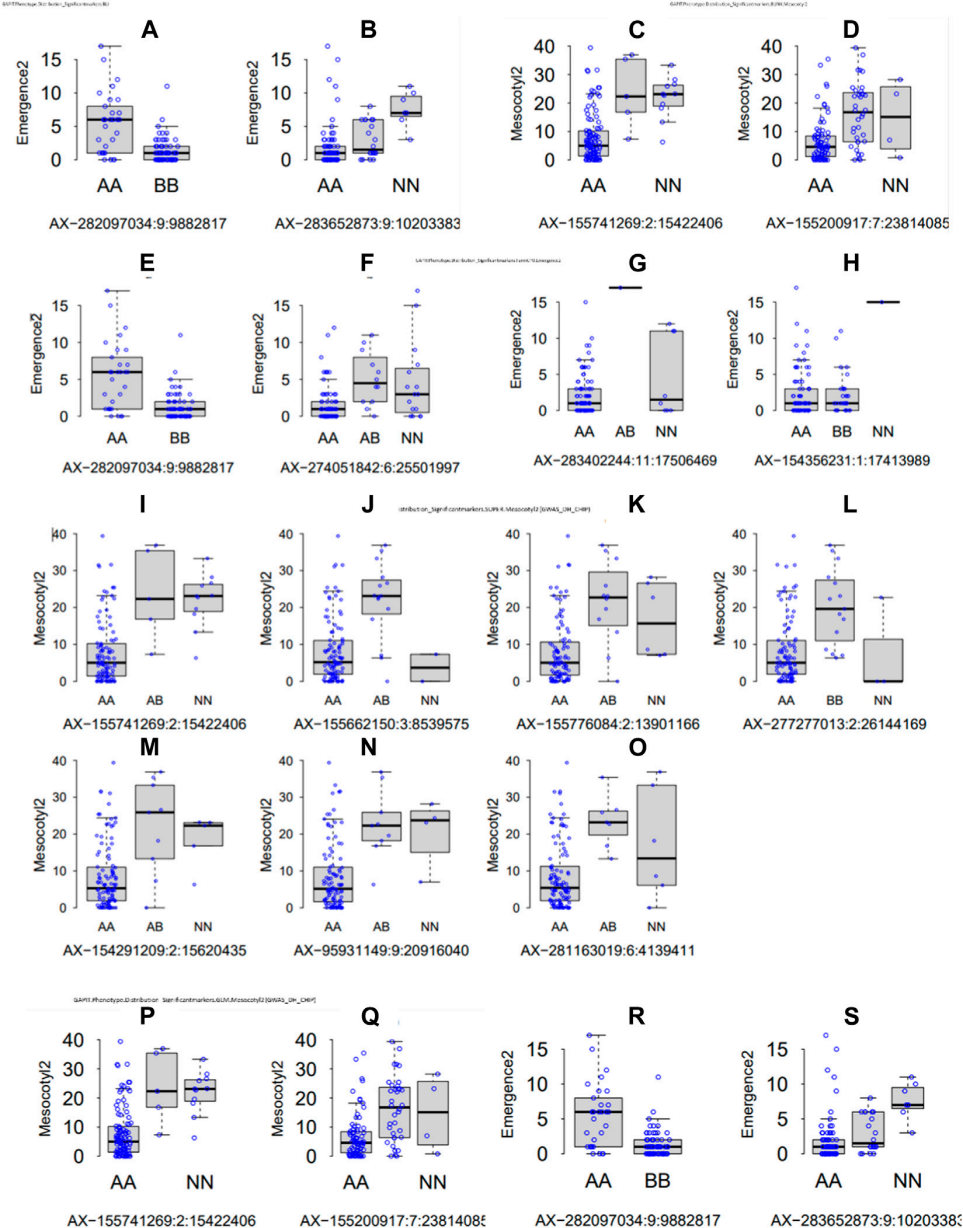
Significant SNP Chip marker-based phenotypic distribution. GAPIT phenotypic distribution based on significant SNP Chip DNA markers for each GAPIT model BLINK **(A–H)** FarmCPU, **(I–O)** SUPER, and **(P–S)** GLM.

Parental recorded differential phenotypes for the selected traits (mesocotyl elongation, seedling emergence, and shoot length), which was reflected in their derived population. In this regard, we performed GWAS with 230,526 SNP Chip DNA markers selected from initial 538,983 markers (after removing those with a high missing rate and heterozygosity above 5%). To explore the statistical powers of proposed GAPIT models to detect significant SNP markers through GWAS, we employed multiple GAPIT models (BLINK, FarmCPU, GLM, and SUPER) to investigate novel genetic loci associated with the control of the traits of interest. In panels Figures 4A–S, we can see the effect of SNP Chip markers on the distribution of phenotypes, grouped by GAPIT models.

**FIGURE 5:**
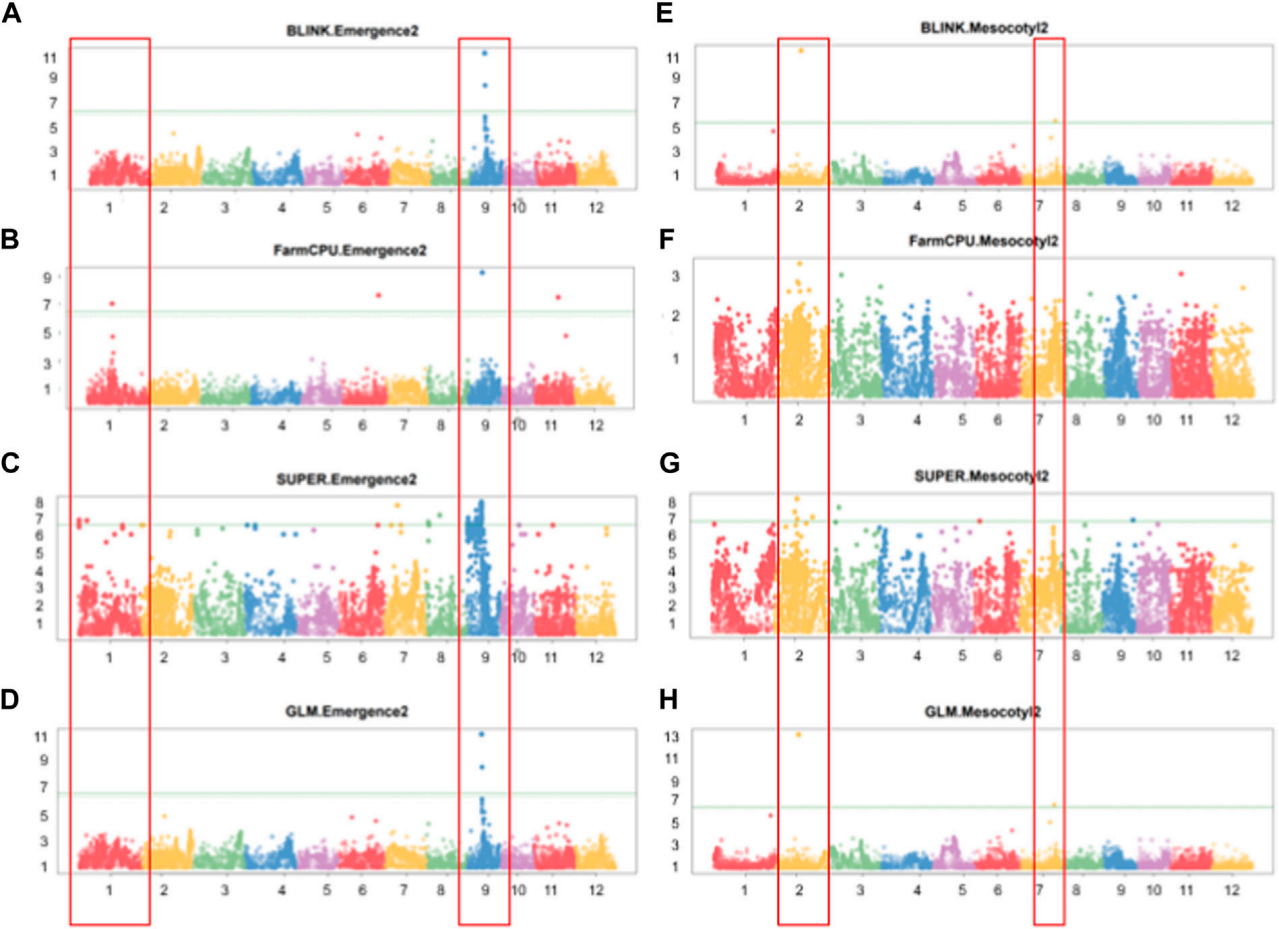
Manhattan plots showing GWAS results for multiple GAPIT models. **(A–D)** Manhattan plots showing significant SNP Chip DNA markers linked to seedling emergence, and **(E–H)** for mesocotyl elongation loci, based on BLINK, FarmCPU, SUPER, and GLM GAPIT models. The frame across the Manhattan plots highlights the detected genetic loci at specific chromosomes, above the genome-wide line.

GWAS results reveal that 13 significant SNP Chip makers are linked to genetic loci controlling mesocotyl elongation or seedling emergence in rice, and are located on 7 chromosomes (Chr) of rice (Figures 5A–H). Eight SNP Chip markers were linked to mesocotyl elongation, found on Chr2, 3, 6, 7, and 9, while those associated with seedling emergence were located on Chr1, 6, 9, and 11 (Table 1). As shown in Table 1, the significant SNP Chip DNA marker AX-282097034 (9882817 bp), linked to the locus controlling seedling emergence on Chr9, was co-detected by several GAPIT models (BLINK, PVE 64.5%; FarmCPU, PVE 13%; SUPER, PVE 3.3%; GLM, PVE 2.1%). The additive effects, −1.41 for AX-282097034 and –3.58 for AX-283652873, indicate that the allele from Milyang352 (*japonica* parent) enhanced the trait value (Table 2). On the same Chr9, five other SNP Chip markers, including AX-283652873 (10203383 bp, PVE 20.2%; additive effect: Milyang352) detected via BLINK, AX-279109564 (9882978 bp, PVE 7.1%, additive effect: Milyang352), AX-155630054 (9922608 bp, PVE 2.9%, additive effect: 93–11), AX-283347022 (6332807 bp, PVE 2.7%, additive effect: 93–11) and AX-117372040 (9910913 bp, PVE 1.8%, additive effect: Milyang352) detected via GLM, were found. Five additional SNP Chip markers with small effects were detected on Chr1, 8, and 9 but their effects were specific to any alleles from either parental lines (Table 2). Likewise, via FarmCPU GAPIT model, other SNP Chip markers, such as AX-154356231 (Chr1:17413989 bp, PVE 21.1%; additive effect: 93–11), AX-274051842 (Chr6: 25501997 bp, PVE 4.2%, additive effect: 93–11), and AX-283402244 (Chr11: 17506469 bp, PVE 36.6%, additive effect: 93–11) were identified.

**TABLE 1:** Significant markers per GAPIT model.

Markers	Chr	Position (bp)	GAPIT model				Trait
AX-154356231	1	17413989	—	FarmCPU	—	—	Emergence
AX-155741269	2	15422406	BLINK	—	SUPER	—	Mesocotyl
AX-155776084	2	13901166	—	—	SUPER	—	Mesocotyl
AX-277277013	2	26144169	—	—	SUPER	—	Mesocotyl
AX-154291209	2	15620435	—	—	SUPER	—	Mesocotyl
AX-155662150	3	8539575	—	—	SUPER	—	Mesocotyl
AX-274051842	6	25501997	—	FarmCPU	—	—	Emergence
AX-281163019	6	4139411	—	—	SUPER	—	Mesocotyl
AX-155200917	7	23814085	BLINK	—	—	—	Mesocotyl
AX-282097034	9	9882817	BLINK	FarmCPU	SUPER	GLM	Emergence
AX-283652873	9	1023383	BLINK	—	—	—	Emergence
AX-95931149	9	20916040	—	—	SUPER	—	Mesocotyl
AX-283402244	11	17506469	—	FarmCPU	—	—	Emergence

**TABLE 2:** Linked markers to *qSEM9-1* and *qMEL2-1* with phenotypic variance explained.

Trait	SNP chip marker	Chr	Position (bp)	*p*-value	MAF	Nobs	Effect	PVE (%)	Allele
Seedling	BLINK								
Emergence	AX-283652873	9	10203383	2E-09	0.22	105	−3.58	20.2	Milyang352
	AX-282097034	9	9882817	2E-12	0.29	105	−1.41	64.5	Milyang352
	FarmCPU								
	AX-154356231	1	17413989	8E-08	0.28	105	1.93	21.1	93–11
	AX-274051842	6	25501997	2E-08	0.19	105	1.42	4.2	93–11
	AX-282097034	9	9882817	5E-10	0.29	105	−1.91	13.0	Milyang352
	AX-283402244	11	17506469	3E-08	0.05	105	2.99	36.6	93–11
	GLM								
	AX-117372040	9	9910913	2E-07	0.31	105	−2.08	1.8	Milyang352
	AX-155630054	9	9922608	1E-07	0.29	105	2.14	2.9	93–11
	AX-283347022	9	6332807	2E-07	0.30	105	2.16	2.7	93–11
	AX-282097034	9	9882817	9E-08	0.29	105	−2.13	2.1	Milyang352
	AX-279109564	9	9882978	1E-07	0.29	105	−2.17	7.1	Milyang352
	SUPER								
	AX-280857006	1	254,877	2E-07	0.28	100	NA	8.3	—
	AX-281773036	8	1471006	2E-07	0.17	100	NA	1.2	—
	AX-154956678	9	1818186	2E-07	0.30	100	NA	1	—
	AX-283647170	9	9090154	3E-07	0.33	100	NA	1	—
	AX-282079312	9	7628982	1E-07	0.30	100	NA	4.1	—
	AX-182198079	9	6846082	1E-07	0.29	100	NA	2.8	—
	AX-283347022	9	6332807	4E-08	0.30	100	NA	3.4	—
	AX-282097034	9	9882817	1E-08	0.29	100	NA	3.3	—
Mesocotyl	BLINK								
elongation	AX-155741269	2	15422406	5.98E-15	0.09	117	11.23	37.5	93–11
	AX-155200917	7	23814085	1.75E-07	0.32	117	3.88	13.8	93–11
	SUPER								
	AX-154291209	2	15620435	2E-07	0.10	112	NA	2.2	—
	AX-155741269	2	15422406	1E-08	0.09	112	NA	6.3	—
	AX-277277013	2	26144169	2E-07	0.14	112	NA	1.3	—
	AX-155662150	3	8539575	5E-08	0.14	112	NA	9.4	—

Because mesocotyl elongation contributes to seedling emergence in rice, we were interested to see genetic loci associated with this trait of interest. As displayed in Tables 1, 2; Figure 5, two SNP Chip markers, AX-155741269 (Chr2: 15422406 bp) detected via BLINK (PVE 37.5%, additive effect: 93–11) and SUPER (PVE 6.3%, additive effect: not specific), and AX-155200917 (Chr7: 23814085 bp) detected via BLINK (PVE 13.8%, additive effect: 93–11) were identified.

Considering that AX-282097034 (Chr9: 9882817 bp) exhibited the highest PVE value (64.5%) for seedling emergence, in addition to being co-detected by four GAPIT models, followed by AX-283652873 (Chr9: 10203383 bp), we were interested to unravel the identity of genes found within the target loci controlling seedling emergence in rice. In the same way, the physical position of the SNP Chip marker AX-155741269 (Chr2: 15422406 bp), co-detected by BLINK and SUPER, and AX-154291209 (Chr2: 15620435 bp) were used to unveil genetic factors associated with the control of mesocotyl elongation in rice. Putative candidate genes (Table 3) for seedling emergence, found within *qSEM9* QTL, were pooled from a region covering 320.6 kbp (with AX-282097034 and AX-283652873 as flanking markers, http://rice.uga.edu/cgi-bin/gbrowse/rice/#search (Chr9:9882817..10203383), accessed on 27 July 2023). Whereas, genes associated with *qMEL2* QTL (Table 4) were pooled from a region (Chr2:15620435..15454000) covering 166.4 kbp.

**TABLE 3:** Putative candidate genes harbored by *qSEM9-1*.

No.	MSU ID	Description (RGAP (Osa1) release 7)	Biological process	Molecular function	Cellular component
Seedling emergence					
1	LOC_Os09g16200	Ankyrin repeat domain-containing protein	—	Protein binding	—
2	LOC_Os09g16240	Ankyrin repeat domain-containing protein	—	Protein binding	—
3	LOC_Os09g16260	dsRNA-binding domain-like, double-stranded RNA binding motif (DSRM) superfamily	—	—	—
4	LOC_Os09g16290	ABC-2 type transporter, putative, expressed, Similar to PDR20	Carbohydrate metabolic process, secondary metabolic process, response to stress, response to biotic stimulus	Transporter activity, hydrolase activity, ATP binding, ATPase activity	Membrane, plasma membrane, plastid, mitochondrion, vacuole
5	LOC_Os09g16330	Pleiotropic drug resistance protein, putative, expressed, Similar to PDR-type ABC transporter 2	Transport, obsolete ATP catabolic process	Transporter activity, hydrolase activity, nucleoside-triphosphatase activity	Membrane
6	LOC_Os09g16380	Pleiotropic drug resistance (PDR-type) protein, putative, expressed, ABC transporter-like domain-containing protein	Transport, response to endogenous stimulus, response to biotic stimulus	Transporter activity, hydrolase activity, ATP binding, ATPase activity	Plasma membrane
7	LOC_Os09g16449	Pleiotropic drug resistance protein 4, putative, expressed, ABC transporter-like domain-containing protein	Transport, response to endogenous stimulus, response to biotic stimulus	Transporter activity, hydrolase activity, ATP binding, ATPase activity	Plasma membrane
8	LOC_Os09g16458	Pleiotropic drug resistance protein 4, putative, expressed, Similar to PDR-like ABC transporter (PDR4 ABC transporter); Similar to PDR20	Transport, response to endogenous stimulus, response to biotic stimulus	Transporter activity, hydrolase activity, nucleoside-triphosphatase activity, ATP binding	Plasma membrane
9	LOC_Os09g16510	WRKY74, WRKY transcription factor 74, expressed	Regulation of transcription, DNA-templated, Biosynthetic process	Sequence-specific DNA binding transcription factor activity	Nucleolus
10	LOC_Os09g16520	Cytochrome b5-like Heme/Steroid binding domain-containing protein, expressed	—	Heme binding	—
11	LOC_Os09g16540	Protein kinase, putative, expressed	Protein modification process; protein phosphorylation	Protein serine/threonine kinase activity, transferase activity, transferring phosphorus-containing groups; carbohydrate binding	Cell
12	LOC_Os09g16550	Ankyrin repeat family protein, putative, expressed, Ankyrin repeat domain containing protein	—	Protein binding	—
13	LOC_Os09g16580	Amidase family protein, putative, expressed	Response to stress, response to biotic stimulus	Hydrolase activity	Endoplasmic reticulum, vacuole, membrane, plasma membrane
14	LOC_Os09g16590	Cysteine-rich receptor-like protein kinase 21 precursors, putative, expressed	Protein modification process	Kinase activity	Plasma membrane
15	LOC_Os09g16650	Legume lectins (L-type (legume-type)) beta domain-containing protein, putative, expressed	Are a highly diverse family of carbohydrate binding proteins that display no enzymatic activity toward the sugars they bind	This family includes arcelin, concanavalinA, the lectin-like receptor kinases, the ERGIC-53/VIP36/EMP46 type1 transmembrane proteins, and an alpha-amylase inhibitor	—
16	LOC_Os09g16700	Legume lectins beta domain-containing protein, putative, expressed	Protein modification process	Kinase activity	Cell

**TABLE 4:** Putative candidate genes harbored by *qMEL2-1*.

No.	MSU ID	Description (RGAP (Osa1) release 7)	Biological process	Molecular function	Cellular component
1	LOC_Os02g26600	ATP binding, related, putative, expressed	Protein metabolic process		Plasma membrane, membrane
2	LOC_Os02g26560	Zinc_ribbon_12 super family; Probable zinc-ribbon domain; This eukaryotic family of proteins has no known function	—	The zinc ribbon domains of the general transcription factors TFIIB and Brf: conserved functional surfaces but different roles in transcription initiation	—
3	LOC_Os02g26550	Ubiquitin carboxyl-terminal hydrolase, family 1, putative, expressed	—	—	—
4	LOC_Os02g26490	NAM-associated super family, No apical meristem-associated C-terminal domain, expressed protein	—	—	—
5	LOC_Os02g26480	OsSCP7 - Putative Serine Carboxypeptidase homologue, expressed	Protein metabolic process	Hydrolase activity	Vacuole
6	LOC_Os02g26460	DUF4371 super family, hAT dimerisation domain-containing protein, putative, expressed	—	—	—
7	LOC_Os02g26440	Prefoldin subunit, putative, expressed	Protein metabolic process	Protein binding	Cytosol
8	LOC_Os02g26430	WRKY42, expressed	Response to stress, response to biotic stimulus	Protein binding, sequence-specific DNA binding transcription factor activity	Nucleus
9	LOC_Os02g26400	Nuclease, EndA/NucM family protein, expressed	—	—	—
10	LOC_Os02g26370	Wiskott-Aldrich syndrome protein family member 2, putative, expressed	—	—	—

We excluded genes with no specific functional conserved domain (hypothetical protein, retrotransposons, etc.). However, genes encoding transposon protein and other domain-containing proteins were included due to their emerging role in plant metabolism.

### Organ-specific validation of *qSEM9* and *qMEL2*-related genes by qPCR

**FIGURE 6:**
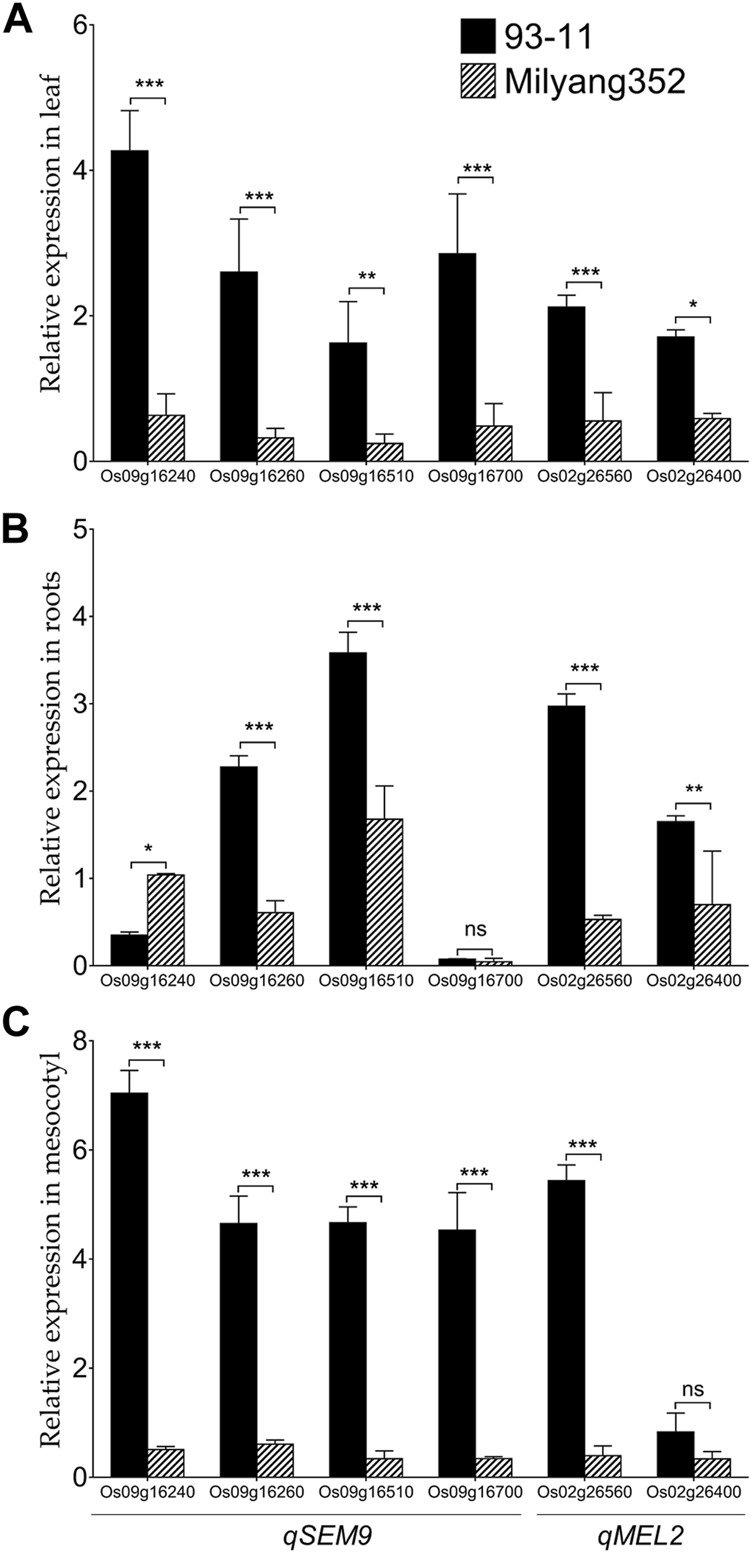
Transcriptional pattern of *qSEM9* and *qMEL2*-related genes in different plant organs of rice. Relative expression of *qSEM9* (*Os09g16240*, *Os09g16260*, *Os09g16510*, and *Os09g16700*) and *qMEL2* (*Os02g26560* and *Os02g26400*) in **(A)** the leaf, **(B)** roots, and **(C)** mesocotyl of parental lines 93–11 (*indica*) and Milyang352 (*japonica*). Black bars are average expression data in 93–11 (*indica*), while bars with hatches are average expression in Milyang352 (*japonica*) background. Error bars are mean values ± SE. ***p < 0.001, **p < 0.01, *p < 0.05, *ns* non-significant.

We were interested to investigate the change in the transcript accumulation level of a set of genes found in *qSEM9* and *qMEL2* regions at basal level. These genes were selected based on the predicted differential coding sequences identified by aligning the *japonica* (Nipponbare rice database: http://rice.uga.edu/#search, accessed on 13 October 2023) (Supplementary Table S1) and *indica* rice database (https://plants.ensembl.org/Oryza_indica/Info/Index, accessed on 13 October 2023) (Supplementary Table S2). qPCR results show that when expressed in the leaf, genes associated with emergence (*qSEM9*) and mesocotyl elongation (*qMEL2*) exhibited a high transcript accumulation level in 93–11 (the *indica* parent with an enhanced emergence rate and longer mesocotyl); while, in Milyang352 (*japonica* parent with low emergence and shorter mesocotyl), the same set of genes maintained a low expression level (Figure 6A). In addition, panel Figure 6B indicates that, except *Os09g16260* and *Os09g16510* (*qSEM9*), and *Os02g26560* and *Os02g26400* (*qMEL2*) which exhibited an enhanced transcriptional level in 93–11 compared to Milyang352, *Os09g16240* (*qSEM9*) expression level was lower in roots of 93–11 but enhanced in Milyang352. Under the same conditions, *Os09g16700* (*qSEM9*) was barely expressed and showed no significant difference between both parents. Likewise, panel Figure 3C shows that the transcript accumulation level of all genes was higher in the mesocotyl of 93–11 than Milyang352, except *Os02g26400* (*qMEL2*), which showed no significant different between the two parental cultivars.

## Discussion

### Seedling emergence is closely related to mesocotyl elongation in rice but not shoot length

Mesocotyl elongation is controlled by a wide range of genetic factors, including a combinational action of plant growth regulators and hormonal signaling and biosynthetic pathway genes, working in a synergetic or antagonistic relationship (Feng et al., 2017). It is widely acknowledged that mesocotyl elongation is essential for seedling emergence in rice (Lee et al., 2017). A study conducted by Wang et al. (2023) found that phytohormones (SLs, CKs, ABA, BRs, IAA, and JA) have a direct influence on mesocotyl elongation by affecting cell division or elongation. Likewise, Liang et al. (2016) supported that an increase in endogenous gibberellin content, which in turn caused changes in cortical microtubule organization, promoted rice mesocotyl elongation. Furthermore, poor emergence of directed-seeded crops is commonly associated with a weak mesocotyl elongation, which may result in poor field occupation and low productivity. In the same way, lines of evidence established that mesocotyl and coleoptile are primarily responsible for the emergence of rice seedlings in deep-sowing and direct-seeding cultivation systems (Wu et al., 2015; Lee et al., 2017). In this regard, we could argue that the observed positive correlation between seedling emergence and mesocotyl elongation on the one hand, and seedling emergence and shoot length, on the other hand, would suggest that the emergence of rice seedlings out of soil from a deep sown cultivation system is likely to depend on the ability of seedlings to develop longer mesocotyls. In turn, a high seedling emergence percentage would be a transition to plant differentiation and the development of aboveground plant parts, which are essential for achieving high productivity and yield potential. In contrast, the non-existing correlation between mesocotyl elongation and seedling shoot length would imply that, after emergence, shoot length might not directly depend on the length of mesocotyl in rice.

### Novel genetic loci for mesocotyl elongation in rice

Mesocotyl elongation and seedling emergence are important traits for the later expression of the yield potential and rice productivity per unit area, and their association has been demonstrated (Lee et al., 2017). Mesocotyl elongation and seedling emergence are determinant factors conditioning the number of individuals present in the field. Since the advent of genome sequencing technologies, several techniques have been used to identify QTLs for mesocotyl elongation in rice, including QTL-sequencing (QTL-seq), GWAS (Lu et al., 2016; Wang et al., 2021) or linkage or association mapping (Edzesi et al., 2023; Feng et al., 2023), and employing diverse mapping populations/germplasm and various DNA marker systems (Huang et al., 2022; Edzesi et al., 2023). Previous studies reported QTLs associated with mesocotyl elongation (*qMEL*) on almost all chromosomes of rice 1–5, 7, 9, 11, 12 (Lee et al., 2012; Lu et al., 2016). According to Wang et al. (2021), genes involved in mesocotyl elongation are also associated with phytohormone metabolic pathways, cell elongation, and cell division. In the present study, eight (8) QTLs for mesocotyl elongation were detected on Chr2 (*qMEL2-1*, *qMEL2-2*, *qMEL2-3*, *qMEL2-4*), 3 (*qMEL3*), 6 (*qMEL6*), 7 (*qMEL7*), and 9 (*qMEL9*), of which number *qMEL2-1* recorded the highest LOD value and was detected by two GAPIT models (BLINK and SUPER). In contrast to previous reports, *qMEL2* is a novel QTL associated with the control mesocotyl elongation in rice. The physical position of *qMEL2* indicates no overlapping region with previously reported mesocotyl-related QTLs in rice (Redona et al., 1996; Cai et al., 2002; Lee et al., 2012; Edzesi et al., 2023). The *qMEL2*, a major mesocotyl QTL explaining nearly 37.5% of the observed phenotypic variance, harbors a set of genes proposed to have diverse molecular functions or be involved in biological processes such as transcription factor activity (*Os02g26430*, encoding a WRKY42 transcription factor, TF), growth and development (*Os02g26490*, no apical meristem (NAM) domain-containing protein, zinc-ribbon domain-containing protein (*Os02g26560*), a Ubiquitin carboxyl-terminal hydrolase (*Os02g26550*), a Serine carboxypeptidase homologue (*OsSCP7*, *Os02g26480*), and a gene encoding the Wiskott-Aldrich syndrome protein (WAS, *Os02g26370*: a conserved domain analysis revealed that this gene has no functional conserved domain. Therefore, it may not be a useful gene (see Supplementary Table S2). Because no conserved domains were detected in Os02g26600 and *Os02g26370* (WAS), their putative functions would be hypothetical.

The role of WRKY TF in plant metabolism has been widely investigated (Finatto et al., 2018), of which we can mention the mediation of the crosstalk between plant growth (through the BR signaling) and abiotic stress (Chen et al., 2017). It was interesting to see that *OsWKRY42* TF, found within the *qMEL2-1* region was earlier reported to suppress *OsMT1d* and induce reactive oxygen species (Han et al., 2014), or mediate resistance to fungal pathogens (Cheng et al., 2015). In addition, Pillai et al. (2018) observed that plants overexpressing *OsWRKY42* TF (described as a cell wall damage-induced TF) enhanced callose deposition (for cell membrane integrity) and anthocyanin content, leading to enhanced salt tolerance in rice, by acting as a negative regulator of the jasmonic acid.

Likewise, members of the NAC family proteins regulate NAM (no apical meristem), regulate drought stress response in rice (Jeong et al., 2010), salt stress in garlic (Wang et al., 2022), heat stress and grain filling in rice (Ren et al., 2021), floral organ identity and lateral organ separation in *Medicago truncatula* (Cheng et al., 2012), pattern formation in embryo and flowers in Petunia (Souer et al., 1996), among others. Mutation in the NAM domain resulted in changes in shoot apical meristem (SAM) development in rice (Prakash et al., 2022) or failure to develop SAM in Petunia embryos (Souer et al., 1996). In the same way, mutant plants lacking the NAM showed abnormal organ differentiation in rice embryos (Nagato et al., 1989).

Similarly, genes encoding the zinc ribbon domain of the general transcription factor TFIIB family play various roles in the transcription initiation events and are highly conserved in plants (Hahn et al., 2000). The TFIIB is essential for RNA Polymerase (Pol) II recruitment (Chen and Hahn, 2003), with which it physically interacts (Elsby and Roberts, 2008) to govern the basal transcription initiation machinery.

The qPCR validation of *qSEM9* and *qMEL2*-related genes revealed differential transcript accumulation patterns of tested genes, except a few. The observed differences in the expression level of target genes between 93–11 (*indica*) and Milyang352 (*japonica*), in leaf, roots, and mesocotyl (see Figures 6A–C), would suggest their differential transcriptional regulation during seedling mesocotyl elongation and seedling emergence. Functional studies are required to elucidate their actual roles during mesocotyl elongation and seedling emergence in rice.

### New genes associated with seedling emergence in rice are identified

As shown in the correlation analysis results, mesocotyl elongation and seedling emergence are closely related. However, as per to the results in Table 3, the novel QTL (*qSEM9*) associated with the control of seedling emergence in direct-seed rice is located on chromosome 9, while the *qMEL2* was detected on chr2. Looking at genes found within the *qSEM9*, based on their annotated functions, they could be grouped into five groups. The first group includes genes associated with protein binding (*Os09g16200* and *Os09g16240*, and *Os09g1652* encoding Ankyrin repeat domain-containing protein and cytochrome b5-like Heme/steroid binding domain-containing protein, respectively). The second and third groups consist of genes proposed to be involved in carbohydrates metabolic process and transport activity [*Os09g16260* encoding an ABC-2 type transporter (*PDR20*); *Os09g16330*, *Os09g16380*, *Os09g16449*, *Os09g16458*, and *Os09g16520* encoding a pleiotropic drug resistance (ABC transporter-like domain), and legume lectins β-domain containing proteins (*Os09g16650* and *Os09g16700*)]. In addition, the majority of genes harbored by the two QTLs are proposed to be involved in abiotic or biotic stress response mechanisms or signaling events (fourth and fifth groups).

### Genomic estimated breeding value of individuals

The advent of molecular breeding techniques and the application of bioinformatics tools in plant breeding have paved new paths toward gene discovery and advancing generations for variety development in a relatively short time. In contrast to conventional breeding, where phenotypic expression of target traits was the major way to assess the genetic merit of individuals, GS or genomic prediction has proven essential to investigate the genetic merit of breeding lines with high accuracy to identify promising lines. This prediction is based on the gBLUP (Zhang et al., 2007) using the MLM approach proposed for GWAS (Zhang et al., 2010), which assesses the genetic potential for a group of individuals derived from the BLUP of group effects in the compressed mixed model and is used as a prediction for all individuals in the group (in the reference or inference cluster). This prediction lies on the assumption that all groups in the reference panel have at least one individual with phenotypic data, and all groups in the inference panel have no individuals with phenotypic data. Hence, the genomic prediction for groups in the inference panel is based on phenotypic ties with their corresponding groups in the reference panel derived from Henderson’s formula (Ferdosi et al., 2019).

## Conclusion and perspectives

Direct-seeded rice is commonly used for several benefits, including cost-effectiveness and seed use efficiency. This study explored the possibility of detecting QTLs using multiple GAPIT models with enhanced statistical power and predicting genomic breeding value in target lines or populations for desired traits. Results identified two major QTLs for mesocotyl elongation and three for seedling emergence in rice. Significant SNP Chip DNA markers tightly linked to the mesocotyl elongation and seedling emergence loci were detected on Chr2 (*qMEL2-1*) and Chr9 (*qSEM9-1*), with 37.5% and 64.5% of PVE, respectively. The *qSEM9-1* (AX-282097034, Chr9: 9882817 bp) was co-detected by four GAPIT models (BLINK, FarmCPU, SUPER, and GLM), while *qMEL2-1* (AX-155741269, Chr2: 15422406 bp) was co-detected by BLINK and SUPER. Furthermore, a high estimated heritability (Mesocotyl elongation: h^2^ = 0.955; seedling emergence: h^2^ = 0.863; shoot length: h^2^ = 0.707) was observed. Genes harbored by *qMEL2-1* and *qSEM9-1* have interesting annotated molecular functions that could be elucidated through functional studies to uncover their roles in mesocotyl elongation and seedling emergence events in rice. Moreover, the presence of genes encoding transcription factors, signaling as well as transport-related genes would suggest that mesocotyl elongation and seedling emergence would involve an active transport of molecules like sugar (a source of energy) and signaling cascade, driven by an active transcriptional regulatory network. Therefore, investigating novel QTLs through GWAS with multiple GAPIT models and high-density SNP Chip DNA marker system could boost the power and accuracy of plant breeding through genome association and prediction, and generate useful data for genomic selection and marker-assisted selection (MAS).

## Data Availability

The original contributions presented in the study are included in the article/Supplementary Material, further inquiries can be directed to the corresponding authors.
