# Supplementary material for: Genome-wide association study (GWAS) with high-throughput SNP chip DNA markers identified novel genetic factors for mesocotyl elongation and seedling emergence in rice (Oryza sativa L.) using multiple GAPIT models
Source: Front Genet. 2023 Nov 20;14:1282620. doi: 10.3389/fgene.2023.1282620 (PMC10694456; doi:10.3389/fgene.2023.1282620)
Supplement: Supplementary file 1 [file DataSheet1.docx]

Table S1. Japonica and indica homologous genes in *qSEM9* region.

| No. | MSU ID | Indica orthologous genes | Description (RGAP (Osa1) Release 7 ) | CDS similarity | Similar report |
| --- | --- | --- | --- | --- | --- |
| 1 | LOC_Os09g16200 | - | Ankyrin repeat domain-containing protein. | - |  |
| 2 | LOC_Os09g16240 | [BGIOSGA029968](https://plants.ensembl.org/Oryza_indica/Gene/Summary?g=BGIOSGA029968;r=9:8677473-8678803;t=BGIOSGA029968-TA;db=core) | Ankyrin repeat domain-containing protein | Deletion 1–816; 2045–2088; 2031 in *indica*; SNP1625[C/T]; SNP1640[C/A]; SNP17938[C/T]; SNP1768[C/A]; SNP1857[A/C]; SNP1879[T/G]; SNP1892[C/T]; SNP1931[T/A]; SNP1939[G/T]; SNP1948[A/B]; SNPs[1996–7]; SNP2001[G/A]; SNP2005[A/G]; SNP2011–12[A/C; C/A] |  |
| 3 | LOC_Os09g16260 | [BGIOSGA029966](https://plants.ensembl.org/Oryza_indica/Gene/Summary?g=BGIOSGA029966;r=9:8696503-8699022;t=BGIOSGA029966-TA;db=core) | dsRNA-binding domain-like, double-stranded RNA binding motif (DSRM) superfamily | Deletion in *japonica* [375–389] |  |
| 4 | LOC_Os09g16290 | - | ABC-2 type transporter, putative, expressed, Similar to PDR20 |  |  |
| 5 | LOC_Os09g16330 | - | Pleiotropic drug resistance protein, putative, expressed, Similar to PDR-type ABC transporter 2 |  |  |
| 6 | LOC_Os09g16380 | - | Pleiotropic drug resistance (PDR-type) protein, putative, expressed, ABC transporter-like domain-containing protein |  |  |
| 7 | LOC_Os09g16449 | - | Pleiotropic drug resistance protein 4, putative, expressed, ABC transporter-like domain-containing protein |  |  |
| 8 | LOC_Os09g16458 | - | Pleiotropic drug resistance protein 4, putative, expressed, Similar to PDR-like ABC transporter (PDR4 ABC transporter); Similar to PDR20 |  |  |
| 9 | LOC_Os09g16510 | BGIOSGA030535 | WRKY74, WRKY transcription factor 74, expressed | SNP246[G/T]; SNP518[C/T] ; SNP639[C/A] ; SNP896[G/C] ; SNP908[A/C] ; SNP968[A/G]; SNP978[C/G] |  |
| 10 | LOC_Os09g16520 | - | Cytochrome b5-like Heme/Steroid binding domain-containing protein, expressed | - |  |
| 11 | LOC_Os09g16540 | [BGIOSGA030536](https://plants.ensembl.org/Oryza_indica/Gene/Summary?g=BGIOSGA030536;r=9:8877528-8880757;t=BGIOSGA030536-TA;db=core) | Protein kinase, putative, expressed | SNP180[T/C]; SNP209[A/G]; SNP218[T/C]; SNP271[A/G]; SNP332[A/G]; SNP371[A/T]; SNP468[A/G]; SNP523[G/A]; SNP907[T/G]; SNP1105[A/G]; SNP1562[C/T]; SNP1722[T/C]; SNP1797] |  |
| 12 | LOC_Os09g16550 | [BGIOSGA029960](https://plants.ensembl.org/Oryza_indica/Gene/Summary?g=BGIOSGA029960;r=9:8881999-8884356;t=BGIOSGA029960-TA;db=core) | Ankyrin repeat family protein, putative, expressed, Ankyrin repeat domain-containing protein | SNP398[A/G]; SNP627[A/G]; SNP1218[C/A]; SNP1267[C/A]; SNPs1933–34[GG/TT]; SNP2056[G/A] |  |
| 13 | LOC_Os09g16580 | - | Amidase family protein, putative, expressed |  |  |
| 14 | LOC_Os09g16590 | - | Cysteine-rich receptor-like protein kinase 21 precursors, putative, expressed |  |  |
| 15 | LOC_Os09g16650 | BGIOSGA029958 | Legume lectins (L-type (legume-type)) beta domain-containing protein, putative, expressed | SNP537[G/T]; SNP1029[T/C] |  |
| 16 | LOC_Os09g16700 | [BGIOSGA030536](https://plants.ensembl.org/Oryza_indica/Gene/Summary?g=BGIOSGA030536;r=9:8877528-8880757;t=BGIOSGA030536-TA;db=core) | Legume lectins beta domain-containing protein, putative, expressed | Deletion 1–9 in *indica*; 440–443; 664–2184 |  |

Table S2. Japonica and indica homologous genes in *qMEL2* region.

| **No.** | **MSU ID** |  | **Description (RGAP (Osa1) Release 7 )** | **CDS similarity** | **Similar reports** |
| --- | --- | --- | --- | --- | --- |
| 1 | LOC_Os02g26600 | [BGIOSGA006509](https://plants.ensembl.org/Oryza_indica/Gene/Summary?g=BGIOSGA006509;r=2:16797842-16801437;t=BGIOSGA006509-TA;db=core) | ATP binding, related, putative, expressed | Deletion in *indica* [1461–63]; SNP921[G/T]; SNP1959[G/A] | No conserved domain identified |
| 2 | LOC_Os02g26560 | [BGIOSGA008170](https://plants.ensembl.org/Oryza_indica/Gene/Summary?g=BGIOSGA008170;r=2:16778753-16781584;t=BGIOSGA008170-TA;db=core) | Zinc_ribbon_12 super family; Probable zinc-ribbon domain; This eukaryotic family of proteins has no known function | Deletion in *indica* [2468–2482] |  |
| 3 | LOC_Os02g26550 | - | Ubiquitin carboxyl-terminal hydrolase, family 1, putative, expressed |  |  |
| 4 | LOC_Os02g26490 | - | NAM-associated super family, No apical meristem-associated C-terminal domain, expressed protein |  |  |
| 5 | LOC_Os02g26480 | - | OsSCP7 - Putative Serine Carboxypeptidase homologue, expressed |  |  |
| 6 | LOC_Os02g26460 | - | DUF4371 super family, hAT dimerization domain-containing protein, putative, expressed |  |  |
| 7 | LOC_Os02g26440 | [BGIOSGA008166](https://plants.ensembl.org/Oryza_indica/Gene/Summary?g=BGIOSGA008166;r=2:16715974-16719313;t=BGIOSGA008166-TA;db=core) | Prefoldin subunit, putative, expressed | Deletion in *japonica* [191–193] |  |
| 8 | LOC_Os02g26430 | [BGIOSGA008165](https://plants.ensembl.org/Oryza_indica/Gene/Summary?g=BGIOSGA008165;r=2:16713199-16714144;t=BGIOSGA008165-TA;db=core) | WRKY42, expressed | SNP70[T/C]; SNP320[A/C] |  |
| 9 | LOC_Os02g26400 | [BGIOSGA006513](https://plants.ensembl.org/Oryza_indica/Gene/Summary?g=BGIOSGA006513;r=2:16700817-16705357;t=BGIOSGA006513-TA;db=core) | Nuclease, EndA/NucM family protein, expressed | Deletion in *japonica* [1-648] |  |
| 10 | LOC_Os02g26370 | [BGIOSGA006516](https://plants.ensembl.org/Oryza_indica/Gene/Summary?g=BGIOSGA006516;r=2:16681557-16684229;t=BGIOSGA006516-TA;db=core) | Wiskott-Aldrich syndrome protein family member 2, putative, expressed | SNP468 [T/G] | No conserved domain identified |
